# German Trial for Holding an Irregular Diploma
*Translated by Arnold Wietfeld.


**Published:** 1886-04

**Authors:** F. Zeitung

**Affiliations:** Berlin


ARTICLE VI.

### GERMAN TRIAL FOR HOLDING AN IRREGULAR DIPLOMA.*

The so-called "American dentist" Henry Sander, used years since the title "D. D. S.,—Sander," who never was in America, showed the government a diploma of the "Nova Anglica Universities of Boston," which he obtained in absentia through an English agent. Sander was condemned to pay by the government fifty dollars. Generally this diploma of "Dr. in absentia" being given out at the name of Philadelphia, but the latter was and many others are at the name of other cities in the United States. The government found out that there don't exist in the United States of America, neither a American University of Philadelphia, nor a Electic Medical College of Pennsylvania, or a Livingstone University of America, or a Nova Anglica Universities of Boston, or a University of South-Ohio of Cincinnati, or a Wisconsin Dental College.

There are still people who buy this kind of diplomas, therefore we only can be very glad that our government acts so strongly against this "Dr. in absentia." The reputation

*Translated by Arnold Wietfeld.

of the better American Institutions can only be ruined by these swindlers, as the numerous and good schools in dentistry of Baltimore, Boston, etc. cannot be made responsible for this kind of business, especially because the better Universities and Colleges require two years study since last year.

Our jury acts very strongly against the so-called "American dentists in absentia."

A short time ago we would mention the sentence of the mechanical dentist Zahntechniker Reseock, who never was in America, but nevertheless called himself M. D., D. D. S.

Since then the dentists "Von Guerard and Grunbaum" had to defend themselves on account of their bought title of American D. D. S.

It was also announced to the Hofzahnarzt, Er Robert Telschow, who likewise never had been in America, and also called himself American D. D. S., that if he should call himself any longer D. D. S., he would be punished according to law.

It has been confirmed by the government that the Doctor manufactory, so-called American University of Philadelphia, has not only nothing to do with the University and the dental colleges of that town, but don't exist at all, but that a swindler, John Buchanan by name, made a good deal of money by selling diplomas of "D. D, S. in absentia;" in consequence of which he was punished to stay in jail several years.

The name of the city of Philadelphia is according to this, not very well liked, but without any reason, because the good institutions of this town cannot be made responsible for this swindle. Berlin:—F. ZEITUNG.

---

